# Mechanisms for change: A theoretical pathway for a school-wide social–emotional learning initiative in an urban middle school

**DOI:** 10.3389/fpsyg.2023.977680

**Published:** 2023-02-15

**Authors:** Gwyne W. White, Danielle R. Hatchimonji, Esha Vaid, Christopher C. Simmons, May Yuan, Angela Wang, Maurice J. Elias

**Affiliations:** ^1^Department of Psychology, Merrimack College, North Andover, MA, United States; ^2^Center for Healthcare Delivery Science, Nemours Children's Hospital, Delaware, Wilmington, DE, United States; ^3^Department of Psychology, The Pennsylvania State University (PSU), University Park, PA, United States; ^4^Rutgers, The State University of New Jersey, New Brunswick, NJ, United States

**Keywords:** SEL, school climate, bullying, discipline, academic achievement, middle school

## Abstract

**Introduction:**

Investment in academic instruction without complementary attention to the social–emotional environment of students may lead to a failure of both. The current study evaluates a proposed mechanism for change, whereby academic achievement occurs as a result of the social–emotional learning environment impacting behavioral (discipline) outcomes.

**Methods:**

We tested the hypothesized model during each year of a 3-year intervention to determine whether the relations among these constructs held potential as a pathway for targeted improvement.

**Results:**

Path analysis for each year demonstrated excellent fit [Year 1: *χ^2^*(19) = 76.16, *CFI* = 0.99, *RMSEA* = 0.05,*TLI* = 0.98; Year 2: *χ^2^*(19) = 70.68, *CFI* = 0.99, *RMSEA* = 0.048, *TLI* = 0.98; Year 3: *χ^2^*(19) = 66.59, *CFI* = 0.99, *RMSEA* = 0.05, *TLI* = 0.98] supporting the theoretical model for change. For each year the effect of the SEL Environment construct on discipline was significant, as was the effect of discipline on Academic Performance. Further, the indirect effect of SEL Environment on Academic Performance was significant across all years.

**Discussion:**

The consistency of these relationships supports the proposed logic model as a potential mechanism for change and has the potential to guide interventions for whole school improvement.

## 1. Introduction

As an institution, schools are often tasked with improving the lives of young people through access to support, resources, and services, in addition to academic instruction. Indeed, research has shown that investment in academic instruction without complementary attention to the social and emotional needs of students may lead to failure in both areas (Aygün and Taşkın, 2022). The transition from the final years of elementary school to the next level of schooling is typically when average academic performance falls, particularly for Black and Latinx students (Felmlee et al., 2018; Seeskin et al., 2018). Given that the completion of high school is a critical predictor of future success and overall well-being (De Witte et al., 2013; Rocque et al., 2017), identifying factors that can be modified to support student academic achievement is a valuable target for intervention research.

Programs under the mantle of Social–Emotional Learning (SEL) have been developed in school settings as a means to promote positive social, emotional, and academic growth. These interventions set out to improve student abilities related to a broad set of social and emotional skills in the domains of self-awareness, social awareness, self-management, relationship skills and responsible decision-making (Weissberg et al., 2015; Dermody et al., 2022). A number of different theories have contributed to the development of SEL programs including models of emotional intelligence (Salovey and Mayer, 1990; Goleman, 1995) and emotional consciousness (Damasio, 1999). Emotional intelligence (EI) posits that cognitive abilities and personal characteristics (e.g., emotional abilities, self-regulatory qualities, characteristics of self-awareness, and social skills) are critical for successful interpersonal and goal-oriented outcomes. Key to this construct is the idea that emotional intelligence is an acquired skill, and thus can be enhanced by training and learning (Kanesan and Fauzan, 2019), making EI focus point for intervention work, particularly in the education setting. Relatedly, the research on consciousness and role the feelings has provided biological evidence for the power and role of emotion identification and interpretation for our ability to successfully self-regulate (Damasio and Carvalho, 2013). In tandem with these developments in our understanding of the important role of emotional skills, school-based-prevention experts and educators developed programmatic guidelines to support educational ‘Social Emotional Learning’ interventions for children and youth (Elias et al., 1997).

The results of meta-analysis and large-scale reviews indicate that SEL interventions can result in positive effects in youth behavior, attitudes, and school performance (Taylor et al., 2017; Murano et al., 2020). When SEL is combined with efforts that foster universal values such as compassion, mutual support, and community service, the degree of distress and disconnection students experience in schools may be reduced (Elias, 2014; Linsky et al., 2018; Wortham et al., 2020). However, the mechanism by which student academic outcomes are improved is complex, shaped by a wide variety of factors both intrinsic to students and existing in their external environment.

Findings from interventions in schools seeking improved academic outcomes indicate that whole school improvement may first begin through a positive change in school culture and climate (Wang and Degol, 2016; Darling and Cook-Harvey, 2018; Hamlin, 2021). A positive social–emotional learning environment can provide an atmosphere of support for students to acquire and grow the individual competencies needed for effective participation in classroom learning and school life. The logic model for change would propose that, as a result of these shifts in environment and expectations, classroom behaviors and peer interactions become less disruptive and more positive resulting in fewer disciplinary referrals (Lacoe and Steinberg, 2018; Reaves et al., 2018). The ultimate outcome of these changes would then be seen in academic improvement at the student and school level. The current study explores the validity of this logic model (i.e., that student perception of their social–emotional learning environment would impact discipline referrals which, in turn, would positively impact academic achievement/grades) in the context of a school-wide effort to improve the social–emotional learning environment of the school.

### 1.1. Social–emotional learning environment

The environment in which students learn comprises a diverse range of categories and characteristics, including relationships between students and staff, the norms and values in the school, promotion of culture and ethnic traditions, and the physical structure of the building (Wang and Degol, 2016; Del Toro and Wang, 2021; Grazia and Molinari, 2021). It has been shown that students’ perceptions of school environment are related to students’ academic achievement (Maxwell et al., 2017; Eugene, 2020; Barksdale et al., 2021), students’ behavior, and students’ decisions to remain in or drop out of school (Gage et al., 2016; Jia et al., 2016). Additionally, research has found that the ability of social–emotional programs to be implemented successfully is related to the culture and climate of the school (Osher et al., 2016). This suggests that, to make a difference in academic achievement, interventions that target academic outcomes must contend with various facets of how students perceive their school environment.

#### 1.1.1. School climate

One aspect that research has identified as key to student perceptions of the school environment, and critical for overall school success, is school climate. Thapa et al. (2013) identified five dimensions of school climate: (1) safety (including social–emotional safety), (2) relationships, (3) teaching and learning, (4) institutional environment, and (5) the school improvement process. Broadly, when students perceive these dimensions of their educational environment positively, the literature indicates a wealth of positive outcomes at both the school and individual level, including an influence on the motivation to learn (Wang et al., 2020); supporting less aggression, violence, and disorder (Bryson and Childs, 2018; O’Connor et al., 2020), and less bullying (Espelage and Hong, 2019). A positive perception of school climate can also mitigate the negative impact of the socioeconomic context on academic success (Eugene, 2020), acting as a protective factor for the learning and positive life development of young people (Lester and Cross, 2015; Steinmayr et al., 2018). A positive school perception of a school’s climate by its students is also linked to better overall psychological well-being (Zullig et al., 2018; Capp et al., 2020; Wang et al., 2020). Additionally, and critical for the logic model of the current study, a positive school climate can lower rates of student suspension (Gage et al., 2016). Thus, perceptions of school climate are a key component of an overall positive social–emotional learning environment.

#### 1.1.2. Bullying

There is evidence to suggest that student perceptions of safety (i.e., prevalence of bullying) may be also key aspect of the social–emotional learning environment, and benefit from evaluation distinct from general school climate. Research has identified that feeling safe from harassment and bullying in the school setting is necessary for the promotion of student learning and development (Bradshaw et al., 2021). In schools where students do not experience the supportive norms, structures, and relationships that promote this sense of safety, students are more likely to experience violence and victimization (Williams et al., 2018). Adolescence is a developmental time period during which peer influence is highly formative and peers have been shown to affect each other’s behavior, including acceptance of bullying (Dahl et al., 2018; Halliday et al., 2021). In settings where bullying is perceived as a normative part of the school environment, evidence suggests that there are higher levels of absenteeism and reduced academic achievement (Kim et al., 2020). In sum, students who perceive their environment as safe from bullying are more likely to succeed both academically and socially (Juvonen et al., 2011; Bouman et al., 2012; Thompson, 2019; Huang, 2022), suggesting that perception of bullying is another key component of students’ social–emotional learning environment.

#### 1.1.3. Peer expectations

Finally, there is evidence to suggest that social-normative expectations, or the expectations one has for the achievements of one’s peers, can have an impact on the learning environment (Bell et al., 2019; Vaid et al., 2023). Peer norms have been found to be an important factor in shaping students’ academic behaviors (Dijkstra and Gest, 2015; Gremmen et al., 2017). Research indicates that students who have positive expectations about their educational attainment develop optimistic ideas about their potential and achieve in accordance with these notions (Anderson et al., 2018; Saadat et al., 2019). Positive educational expectations are not only critical for promoting achievement, but these expectations may also be a protective asset for vulnerable, at-risk youth (Herrenkohl et al., 2012; Gerard and Booth, 2015; Stoddard and Pierce, 2015; Brumley et al., 2017). While self-expectations are valuable to understand, social-normative expectations may assess a similar construct while reducing potential biases (self-serving bias theory; Miller and Ross, 1975; Shepperd et al., 2008) and provoke students to also think about potential environmental support and barriers (Vaid et al., 2023). In fact, Sommerfeld (2016) found that social-normative expectations explained educational outcomes above and beyond accounting for self- and parental-expectations. Indeed, research has found that group beliefs or attitudes about academic achievement may have a more substantial influence on academic achievement than expectations about oneself (Bell et al., 2019). These findings suggest that the social-normative expectations students hold may influence student behaviors as well as academic outcomes, and may be another core component of a students’ perceptions of their social–emotional learning environment.

### 1.2. Discipline

There is evidence to suggest that, as a result of improvement in student perceptions of the environment and increased social–emotional skills, classroom behavior and instruction can become less disruptive and more positive (Lacoe and Steinberg, 2018; Reaves et al., 2018). This is particularly important in light of the finding that exclusionary school discipline rates in the United States are high, with nearly 2.7 million K-12 students received one or more out-of-school suspensions and over 100,000 students expelled (Gerlinger et al., 2021). Disciplinary actions also have been found to be tied to the race of the student, with racially minoritized students disproportionately affected (Skiba et al., 2011; Anyon et al., 2014; Anyon et al., 2018; Cruz et al., 2021). Notably for the current study, during the middle school grades, there appears to be an increase in both disciplinary rates and racial disparities in discipline and achievement (Skiba et al., 2011; Anyon et al., 2014; White et al., 2016; Gerlinger et al., 2021). Literature further, and unsurprisingly, indicates that high discipline rates tend to be related to negative academic and behavioral outcomes (Anderson et al., 2019; Sorensen et al., 2021). Critical for our understanding of how student perceptions can influence student behaviors, there is also evidence that repeated discipline referrals may trigger a cycle of negative adult-student interaction and may contribute to a student’s psychological disengagement (Gregory et al., 2021). The environment created by the teacher and the school can thus be seen as in a cycle with negative student behavior, whereby students are apt to act out in environments where they feel disrespected and disengaged, and teachers’ response (e.g., discipline referrals) further that alienation (Cook et al., 2018). Conversely, the proposed change model here suggests that, when students feel a positive connection to their school environment, it serves to make them less likely to engage in acting out behavior, and teachers are, in-turn, less likely to respond harshly to minor perceived infractions supporting a cycle of support and engagement (Valente et al., 2019). Thus, a model of interaction could be proposed in either direction – does the students’ perception of their social–emotional learning environment impact discipline, or does discipline impact the perception of the social emotional learning environment? Thus, our study also sought to explore an alternative path between factors, whereby discipline is the first in the cascade, rather than environment. Regardless of direction, however, the evidence suggests that overall school improvement may be related to improving student behaviors, as indicated by disciplinary referrals.

### 1.3. Student academic achievement

The challenge for American public education is to improve student achievement both broadly and, specifically, for those deemed in need of additional educational support. The importance of academic achievement is long-term and self-reinforcing, as academic success confers many long-term benefits. Indeed, research has consistently found that individuals with higher levels of education are less likely to be unemployed and more likely to earn higher incomes than those with lower levels of education (U.S. Census Bureau, 2019).

Academic achievement has been found to be related to a significant number of factors, which have also been historic targets for intervention. For example, the affective qualities of teacher–student relationships have been found to impact students’ as well as teachers’ school engagement and achievement (Roorda et al., 2011; Spilt et al., 2011). Student perceptions of competence and relatedness have also been linked to academic outcomes, particularly in the context of students with social and behavioral difficulties (Olivier et al., 2020; Buzzai et al., 2021). Additionally, teachers that demonstrate higher levels of professional competence have been found to engage in more effective teaching, resulting in improved student learning (Fauth et al., 2019; Kyriakides et al., 2020). At the student level, factors such at childhood intelligence (McCoach et al., 2017), executive functioning (Samuels et al., 2016), and perseverance/grit (Credé et al., 2017) all have an impact on academic achievement. In academic achievement outcomes and interventions, the literature suggests there are many pathways to success.

Of concern regarding issues of equity, is that immigrant, and racial/ethnic minoritized children from low-income families face greater barriers to academic success resulting in reduced chances to earn a high school diploma in comparison to their more affluent, White peers (McKinley Yoder et al., 2022). Further, and related, teacher perceptions of children’s achievement, whether accurate or not, impact students’ grades and scores on standardized achievement tests (Jussim et al., 2009; Liang and Zhang, 2009; Zajda, 2021). These expectancy effects appear strongest for non-White and for low SES youth (McKown and Weinstein, 2008; Fitzpatrick et al., 2015), which may explain the increasing impact that race has on achievement scores from elementary to middle and high school (White et al., 2016). This achievement gap was exacerbated during the COVID-19 pandemic as communities of color continue to face disproportionate detrimental health and economic impacts (U.S. Bureau of Labor Statistics, 2019). Unfortunately, low resourced schools, (e.g., those with high-poverty populations) have historically experienced challenges in implementing effective interventions aimed at achievement due to range of factors (Herman, 2012; Strunk et al., 2016).

Evidence suggests that the social and emotional needs of students are an important component of this overall achievement goal, if not a gatekeeper of academic progress (Corcoran et al., 2018). Thus, it is critical to approach the academic needs of all student populations from a strengths-based, whole-child approach. A youth mindset of perseverance, a construct that has been empirically linked to academic success (Farrington et al., 2012; Yeager and Dweck, 2012), can be fostered in a supportive social–emotional learning environment where interpersonal resilience is scaffolded by intrapersonal engagement (Corcoran et al., 2018). The ongoing and long-term consequences of the COVID-19 pandemic suggest that it is even more important than ever to explore ways to support student resilience and academic achievement.

### 1.4. The present study

Pathways to sustained improvement in academic achievement are a multidimensional and multistep process, and the mechanisms by which change can occur benefit from validation. The present study seeks to evaluate the theoretical model for change, that hypothesized that, by addressing the social–emotional learning environment, student behaviors would improve, resulting in fewer disciplinary referrals and, ultimately, allowing for overall improved academic achievement. These factors were explored because they were employed by a 3-year school-based intervention (“School of Character”) and the current study seeks to assess the value of the model for change imbedded within that active intervention. Our study additionally tested an alternative pathway, to see if the pathway for change alternatively occurred by addressing student problem behaviors improved the social–emotional learning environment, allowing for overall improved academic achievement.

The current study explores the relationships among the domains targeted by the School of Character program to provide support for the logic model proposed and implemented by this intervention. The theory proposed was that, by positively impacting the social–emotional learning environment, there would be a resulting cascading impact on academic achievement. The expectation was to see an impact on disciplinary referrals as function of this pathway. In order to understand the success or failure of SEL intervention programs like the School of Character program, it is critical to evaluate if the proposed mechanisms of change, the pathways by which the intervention hopes to achieve outcomes, are valid. The current study explores the structural pathways between the target constructs to test the underlying theory for the hypothesized change model proposed in the School of Character intervention, as well as an alternative pathway where discipline impacts the social–emotional learning environment. Analyses, therefore, focused on a cross-sectional analysis of each year of the 3 years of the intervention to assess if the underlying theoretical model holds true across time and student population, irrespective of external factors, including intervention impacts.

## 2. Method

Data for this project were drawn from a 3-year school improvement (School of Character) initiative that assessed school climate and indicators of the school’s functioning in an urban middle school in New Jersey. This study was approved by the University Institutional Review Board.

### 2.1. Participants

This urban middle school generally reflected a student population of approximately 1,300–1,400 students, grades 6 through 8. Students were majority Latinx. The student population also reflected a lower income lower socio-economic status based on percent of students qualifying for free or reduced lunch (a proxy variable for parent income due to the federal income standards required for student receipt of free/reduced lunch price). During Year 1, the mean age of the students at the time of the survey was 12.84, SD = 1.16 (range = 10–16), at Year 2, the mean age of the students was 12.83, SD = 1.12 (range = 11–17), and at Year 3, the mean age of the students was 12.67, SD = 1.02 (range = 11–16). Demographic data for the school at each year of the study are presented in Table 1. Across all 3 years, the school population consistently reflected a majority Latinx population (Year 1: 88%; Year 2: 90%; Year 3: 92%). The student population also predominately met federal criteria to receive Free Lunch (Year 1: 90%; Year 2: 91%; Year 3: 91%). Due to the homogeneity of these results, further analyses by ethnicity and income-status were not conducted.

**Table 1:** Demographics characteristic by year.

	Year 1		Year 2		Year 3	
	N	%	N	%	N	%
Grade						
6^th^	431	37.8%	435	35.8%	433	42.8%
7^th^	390	34.2%	413	34.0%	311	30.7%
8^th^	319	28.0%	367	30.2%	268	26.5%
Gender						
Male	587	51.5%	633	52.1%	498	49.2%
Female	553	48.5%	582	47.9%	514	50.8%
Lunch status						
Full price	57	5.0%	51	4.2%	46	4.5%
Reduced	60	5.3%	55	4.5%	44	4.3%
Free	1,023	89.7%	1,109	91.3%	922	91.1%
Classification (LEP or IEP)						
None	890	78.1%	928	76.4%	741	73.2%
Support	250	21.9%	287	23.6%	271	26.8%
Ethnicity (according to School)						
White	7	0.6%	3	0.2%	2	0.2
Black	121	10.6%	107	8.8%	79	7.8
Hispanic	1,005	88.2%	1,097	90.3%	926	91.5
Asian	5	0.4%	5	0.4%	2	0.2
Multi-Ethnic	2	0.2%	3	0.2%	3	0.3
Latinx						
Not Latinx	135	11.8%	118	9.7%	86	8.5
Latinx	1,005	88.2%	1,097	90.3%	926	91.5%
Country of origin						
Not US Born	269	23.6%	267	22.0%	207	20.5%
US Born	871	76.4%	948	78.0%	805	79.5%
Total	1,140		1,215		1,012	

### 2.2. Procedures

The three-year SEL “School of Character” intervention engaged a whole-school intervention model, including several initiatives to impact all students and staff in the school. The methodology of the project followed community-based participatory action research guidelines. The district targeted by this intervention had one of the lowest graduation rates in the state (under 60%) and reading and math testing scores below the 15th percentile in the state. The school in which the intervention was implemented was designated as a “priority” school, an iteration of language used to denote a “failing” school and was publicly known as the “worst” middle school in its entire county. Preliminary work by the School of Character intervention identified that both the culture/climate of the school and the number of disciplinary incidents/referrals were of significant concern to teachers and administration. The intervention program therefore, co-developed with school staff, was intended to help build the positive adult climate and then, by being a source of both engagement and value to students, improve students’ perceptions of the climate and greater engagement in the school through reduced disruptive behaviors and increase attention to academics. Research team members partnered directly with administrators and teachers to summarize discipline data and infuse SEL practices into the school discipline system, particularly in the context of In-School Suspension. To simultaneously address staff culture and climate and student discipline concerns, the research team and school staff formed several staff-led committees. One overarching “Climate and Culture” committee, led by one of the school’s guidance counselors, met regularly to set an overall strategy for improving school climate and culture, using aggregated school data (e.g., climate surveys, discipline data). Initiatives included opportunities to provide positive feedback amongst staff in a monthly newsletters and hosting community-building events. Subcommittees included a team tasked with the co-creation of an SEL curriculum implemented in daily advisory periods. This team also monitored and supported implementation of the daily curriculum, with one-on-one coaching and modeling for teachers who requested support. Additional subcommittees focused on youth empowerment initiatives.

Data for the current study were collected as part of that school-wide intervention during the 2012–2013, 2013–2014, and 2014–2015 academic years. Survey data were collected from all students during the Fall and Spring for all 3 years, with the exception of Fall of the 2014–2015 school year. Due to administrative concerns regarding the logistics and time–cost associated with a school-wide survey, student data were only collected for 6^th^ graders in the Fall of 2014. In the Spring of 2015, survey data was again collected school-wide for all students. Students and their parents were given the opportunity to opt out of the data collection both through a passive consent form sent home to the parents and an assent form given to the students prior to survey administration. Less than 1% of students or parents opted out. In addition, school records were reviewed to obtain student demographic and academic data.

### 2.3. Measures

#### 2.3.1. Survey data: School climate

School climate was measured using an adaptation of the School as a Caring Community Profile-II (Lickona and Davidson, 2003), a 42-item measure of perception of school climate. In order to reduce item redundancy and administration time, 22 items from the original measure, with factor loadings below 0.40 or cross factor loadings, were eliminated. The shortened questionnaire consisted of 20 items, for which students rated their agreement on a 5-point Likert Scale, ranging from “Disagree A LOT!” to “Agree A LOT!” Survey included items evaluating student perception of their peers, with questions such as: “students treat classmates with respect;” perception of their teachers, with questions such as: “Teachers in this school like to come here;” and student perception of the student-teacher relationships, with questions such as: “Teachers are unfair in their treatment of students.” A total score for this scale was created by summing the items with a higher score equating a more positive sense of school climate. At the time of this study, the SCCP-II was the only empirically supported scale with parallel items for all grade levels, an important consideration to the school district in adopting a school climate measure. Cronbach alphas for each of 3 years, Fall and Spring, ranged from 0.83 to 0.88, suggesting good reliability.

#### 2.3.2. Survey data: Perceptions of bullying

Student perceptions of bullying were evaluated using an 8-item scale created by the research team. The items were developed based on existing assessments of bullying (Williams and Guerra, 2007; Swearer et al., 2010; Espelage and Hong, 2019). Each item used a 5-point Likert Scale, ranging from “Disagree A LOT!” to “Agree A LOT!” Survey items included questions evaluating students’ sense of general school safety, including items such as: “Students at this school feel safe,” and ‘When students see another student being picked on or put down, they try to stop it.” Questions also assessed student perceptions of individual level bullying with items such as “Students are often bullied or teased in my school” and “My classmates use computers, videos, smart phones, and other technology to harass other students.” Negative items were reverse coded and a total score for this scale was created by summing the items with a higher score, indicating a more positive perception of school safety (less bulling). Cronbach alphas for each of 3 years, Fall and Spring, ranged from 0.69 to 0.75.

#### 2.3.3. Survey data: Social normative expectations

Social-Normative Expectations (SNE), asked students to rate their peers on six items adapted from a study on educational attainment in the Chicago Public Schools (Ou and Reynolds, 2008) and was evaluated as a construct using pilot data from the current project (Bell et al., 2019). Declarative statements were rated on a 5-point Likert Scale ranging from “Disagree A LOT!” to “Agree A LOT!” Questions included items such as: “In the future, most students from this school will graduate from high school” and “In the future, most students in this school will have a happy family life.” A single total score for this scale was created by summing the items. Higher scores indicated more favorable ratings of social-normative expectations, i.e., a belief that peers expected to attain success across the six areas. Cronbach alphas for each of 3 years, Fall and Spring, ranged from 0.87 to 0.92.

#### 2.3.4. Discipline referrals

Disciplinary data for students were provided by the school. Examples of discipline referrals include such minor misbehavior as ‘dress code violation,’ ‘in the halls without a pass,’ and ‘tardies to class,’ as well as major discipline referrals such as ‘harassment/bullying,’ ‘threatening a staff member/student,’ and ‘serious disruptive/inappropriate behavior.’ In Year 1, the total number of discipline referrals per student ranged from 0 to 121 with a Mean of 5.95 (SD = 13.05; Median = 1.0) with approximately 43% of students evidencing no referral. In Year 2, the total number of discipline referrals ranged from 0 to 134 with a Mean of 8.10 (SD = 15.11; Median = 2.0) with approximately 31% of students evidencing no referral. In Year 3, the total number of discipline referrals ranged from 0 to 65 with a Mean of 2.90 (SD = 5.92; Median = 1.0) with approximately 44% of students evidencing no referral. In order to identify the sample into a relatively even distribution, discipline referrals were recoded into a 0–5 scale for each year, with 0 coded as no discipline referrals, 1 coded as a single discipline referral, 2 coded as 2–3 discipline referrals, 3 coded as 4–7 discipline referrals, 4 coded as 8–20 discipline referrals and 5 coded as greater than 21 discipline referrals (see Table 2).

**Table 2:** Discipline referrals by year.

	Year 1	Year 2	Year 3
	*n*(%)	*n*(%)	*n*(%)
No Discipline referrals	484(42.5%)	375(30.9%)	448(44.3%)
1 Discipline referral	145(12.7%)	180(14.8%)	182(18.0%)
2–3 Discipline referrals	151(13.2%)	168(13.8%)	158(15.6%)
4–7 Discipline referrals	126(11.1%)	161(13.3%)	114(11.3%)
8–20 Discipline referrals	141(12.4%)	185(15.2%)	85(8.4%)
Greater than 21 Discipline referrals	93(8.2%)	146(12.0%)	25(2.5%)

#### 2.3.5. Academic achievement

Academic grades were obtained from official school records and used in their numeric form, rather than as letter grades (i.e., 95, not “A”), in order to preserve the continuous nature of the data. Academic achievement was measured using the mean of the four quarters for each of the four core subject areas (Language Arts, Math, Science, and Social Students). An average final overall grade was created from these grades that represented a synthesis of the year’s academic efforts. Grades, rather than standardized tests, were used as the indicator of academic achievement because of literature supporting grades as better predictors of high school graduation, college performance, and longer-term life outcomes than standardized tests (Geiser and Santelices, 2007). Students who received a grade in 3 out of 4 quarters for 3 out of 4 core subject areas (Language Arts, Math, Science, and Social Students) were considered to have a valid final grade for data analysis. Academic achievement data by year of study are presented in Table 3.

**Table 3:** Academic achievement data by year.

	Year 1		Year 2		Year 3	
	M	SD	M	SD	M	SD
Year 2012–2013						
Language Arts	75.63	9.81	--	--	--	--
Mathematics	74.67	10.84	--	--	--	--
Science	77.75	9.80	--	--	--	--
Social Studies	78.15	10.36	--	--	--	--
Overall Grade	76.55	8.86	--	--	--	--
Year 2013–2014						
Language Arts	--	--	75.43	9.26	--	--
Mathematics	--	--	75.99	10.32	--	--
Science	--	--	77.47	9.82	--	--
Social Studies	--	--	78.30	10.83	--	--
Overall Grade	--	--	76.80	8.78	--	--
Year 2014–2015						
Language Arts	--	--	--	--	75.22	9.13
Mathematics	--	--	--	--	75.15	10.77
Science	--	--	--	--	77.82	9.76
Social Studies	--	--	--	--	75.98	9.53
Overall Grade	--	--	--	--	76.04	8.43

#### 2.3.6. Covariates

In order to control for the known effects of demographic information on academic achievement, we explored four covariates, grade level, gender, if a student received academic support (Individualized Education Plan or Limited English Proficiency) and country of origin (US born or not). Data were obtained from official school records. These covariates were explored due to their documented impacts on academic achievement and discipline (e.g., Porter, 2000; Hubbard, 2005; La Salle et al., 2013; Moreno and Segura-Herrera, 2013; Santiago et al., 2014; Gašević et al., 2016; Morris and Perry, 2017; Daily et al., 2019).

### 2.4. Missing data

Students without demographic information from the school, who completed less than 3 core classes (language arts, mathematics, science, social studies) were excluded from analysis. Further, the analysis sample was reduced to those who had responded to, at minimum, half of items on each of the 3 social–emotional learning environment survey measures (Climate, Bullying, SNE). Finally, the preferred data point for survey analysis was spring, however, to reduce the bias from missing survey data, if a student had completed a fall survey but not spring, the fall data was substituted (see Table 4). Due to having Fall of 2014 student data for 6^th^ grade students only, any 7^th^ or 8^th^ grade students missing Spring of 2015 survey data were not included in analyses for that year. As a result, a relative reduction in analysis sample size (approximately 100 less students than previous years) occurred.

**Table 4:** Student perceptions of social emotional learning environment by year.

	Year 1			Year 2			Year 3		
Student-reported measures	*n*	*M*	*SD*	*n*	*M*	*SD*	*n*	*M*	*SD*
School climate									
Fall*	984	63.04	12.88	910	64.72	12.33	414	72.02	12.31
Spring	1,044	59.15	11.78	1,154	62.98	11.67	909	67.03	12.67
Analysis sample	1,140	59.30	11.92	1,215	64.13	12.06	1,012	67.36	12.73
Perceptions of bullying									
Fall*	979	24.02	5.73	896	25.30	5.79	390	27.53	5.78
Spring	1,039	23.32	5.65	1,142	25.38	5.56	947	27.22	5.72
Analysis sample	1,140	23.30	5.62	1,215	25.33	5.55	1,012	27.16	5.72
Social normative expectations									
Fall*	986	19.38	5.70	904	20.38	5.51	403	22.47	5.38
Spring	1,046	18.82	5.51	1,153	19.44	5.21	977	20.98	5.46
Analysis sample	1,140	18.89	5.50	1,215	19.49	5.22	1,012	20.98	5.49

* Fall of Year 3 was completed by 6th grade students only.

### 2.5. Data analyses

Preliminary analyses were conducted to understand the relationships among study variables. All predictor variables were standardized before being entered into the modeling analyses. T-tests and One-Way Analysis of Variance were run to examine differences between the potential demographic variables (gender, grade level, country of birth and if the student received support such as a 504 plan or LEP) and predictor (school climate, social-normative expectations, bullying) and outcome variables (grades).

We tested the hypothesized pathway model, whereby student perception of their social–emotional learning environment (climate, bullying and social normative expectations) impacted discipline referrals, which in turn impacted final grade over three timepoints. This model involved three points (Year 1, Year 2, and Year 3 of the School of Character intervention) examined independently rather than a change model assessing the impact of the intervention on the constructs from Year 1 to Year 3. While the School of Character intervention proposed to improve academic achievement by its implementation, the current study does not explore the efficacy of that program in a longitudinal model of change. Our hypothesis is that the theoretical mechanism of change employed by this intervention (that the social–emotional learning environment impacts discipline which impacts academic achievement) has conceptual validity, with the constructs and variables interacting in such a way that positive academic outcomes could theoretically result from improvement in student perceptions of the social–emotional learning environment. The model explored here is that the proposed pathways between variables are significant, and that another model does not better explain the relationship between the study variables. The efficacy of the School of Character intervention itself must be examined separately so as to accuracy reflect the strengths, weaknesses, successes and failures of a program implemented within a complex community sample and academic system. If the underlying theoretical model for change utilized by the School of Character program has support, future intervention work can then potentially utilize the theoretical model proposed here.

Covariates were not included in analysis model as demographic factors were not predicted to differentiate the proposed mechanism for change being tested. All variables were entered into the sample model and path analysis was used to test a “structural model” (Cohen et al., 1993). For all models, the continuous variables were centered to reduce multicollinearity. Path analysis, while similar to regression analyses, is considered to be more powerful as it examines linear relationships with path coefficients calculated simultaneously for all endogenous variables, rather than sequentially as in multiple regression models, as well as accounting for measurement error. Path analysis has been used to support identifying causal relationships, however, the current study design is not a causal model. Our analyses seek to identify whether the hypothesized path relationships between the study variables were significant, or a different path model would offer a better explanation. Both direct and indirect effects are estimated in the structural model (Kline, 2011). Good fitting models generally have non-significant chi-square values, TLI at or above 0.90, CFI at or above.95, and RMSEA at or below.06. Parameters were established as statistically significant with alpha <0.05. All preliminary analyses were conducted using SPSS software, version 27 (IBM Corporation, 2021) and the modeling analyses was conducted with AMOS software (Arbuckle and Wothke, 2006).

## 3. Results

### 3.1. Preliminary analyses

Pearson’s correlations were conducted between academic achievement variables (i.e., LA, Math, Science, Social Studies, and Overall Grade) across all 3 years of the study. The relationships between all achievement variables were established to be highly significant and generally consistent across the 3 time points (*r* = 0.62–0.77; *p* < 0.001; See Table 5). Greater variability was identified in the relationship between academic achievement and other study variables (i.e., discipline, climate, bullying, and social normative expectations) across time points (see Table 6; Supplementary material). Notably, academic achievement and discipline referrals were consistently, significantly negatively correlated across all 3 years (*r* = −0.67–−0.51; *p* < 0.001), and all SEL environment measures were significantly positively correlated (*r* = 0.41–0.66; *p* < 0.001). Additionally, the number of discipline referrals and student perceptions of school climate were consistently, significantly negatively related across all 3 years [*r*(1140) = −0.16–−0.12; *p* < 0.001].

**Table 5:** Pearson’s correlations among academic achievement variables.

	1			2			3			4		
	Year 1	Year 2	Year 3	Year 1	Year 2	Year 3	Year 1	Year 2	Year 3	Year 1	Year 2	Year 3
1. LA	--	--	--									
2. Math	0.70***	0.63***	0.62***	--	--	--						
3. Science	0.66***	0.68***	0.67***	0.67***	0.71***	0.67***	--	--	--			
4. Social Studies	0.66***	0.68***	0.72***	0.64***	0.62***	0.57***	0.70***	0.77***	0.68***	--	--	--
5. Overall Grade	0.87***	0.85***	0.87***	0.87***	0.85***	0.84***	0.87***	0.90***	0.88***	0.87***	0.87***	0.86***

*** *p* < 0.001.

**Table 6:** Pearson’s correlations among continuous study variables.

	1			2			3			4		
	Year 1	Year 2	Year 3	Year 1	Year 2	Year 3	Year 1	Year 2	Year 3	Year 1	Year 2	Year 3
1. Overall grade	--	--	--									
2. Discipline	−0.57***	−0.61***	−0.51***	--	--	--						
3. Climate	0.04	0.08**	−0.08*	−0.12***	−0.13***	−0.16***	--	--	--			
4. Bullying	0.06	0.04	0.08*	−0.10*	−0.05	−0.12***	0.61***	0.47***	0.66***	--	--	--
5. SNE	−0.07*	−0.03	0.00	−0.08**	−0.01	−0.05	0.56***	0.47***	0.60***	0.41***	0.42***	0.58***

* *p* < 0.05;

** *p* < 0.01;

*** *p* < 0.001.

Independent t-tests and ANOVAs were also conducted to examine the impact of demographic covariates on academic achievement and discipline (Table 7; Supplementary material) and on measures of the SEL environment (Table 8; Supplementary material). Grade level appeared to have some impact on measures of the social–emotional learning environment, with 6^th^ graders evidencing a better perception of school climate across all 3 years [*F*(2,1,137) = 7.11, *p* = 0.001; *F*(2,1,212) = 44.90, *p* < 0.001; *F*(2,1,009) = 17.41, *p* < 0.001 respectively]. Grade level appeared to have a varying impact on perceptions of bullying, with a significant relationship during year 1 and 2 [*F*(2,1,137) = 3.88, *p* = 0.021; *F*(2,1,212) = 4.71, *p* = 0.009 respectively] with 7^th^ graders reporting the least positive perceptions of student bullying culture during both years. Positive social normative expectations were most consistently reported by 6^th^ graders across all 3 years [*F*(2,1,137) = 25.81, *p* < 0.001; *F*(2,1,212) = 15.28, *p* < 0.001; *F*(2,1,009) = 9.30, *p* < 0.001 respectively]. Gender had no impact on any social emotional learning variable across any of the 3 years (see Table 8; Supplementary material).

**Table 7:** Impact of demographic covariates on overall grade and discipline.

	Overall grade						Discipline					
	Year 1		Year 2		Year 3		Year 1		Year 2		Year 3	
	*M*	*SD*	*M*	*SD*	*M*	*SD*	*M*	*SD*	*M*	*SD*	*M*	*SD*
Grade												
6^th^	77.60**	8.97	78.17***	8.02	76.56	8.58	1.53	1.77	1.86	1.76	1.39*	1.48
7^th^	75.92	8.15	75.31	8.71	75.29	8.41	1.89**	1.78	2.19*	1.85	1.31	1.47
8^th^	75.90	9.41	76.84	9.44	76.09	8.18	1.44	1.62	2.07	1.74	1.10	1.38
Gender												
Male	74.55	8.80	75.00	8.67	73.57	8.02	1.80***	1.78	2.24***	1.78	1.56***	1.54
Female	78.68***	8.42	78.75***	8.48	78.44***	8.14	1.44	1.68	1.81	1.78	1.03	1.32
Classification												
None	76.67	9.13	77.23**	8.80	76.25	8.82	1.58	1.76	1.92	1.79	1.18	1.40
Support	76.11	7.79	75.39	8.55	75.47	7.24	1.79	1.67	2.38***	1.76	1.59***	1.56
Country of Origin												
Not US	76.15	8.89	76.89	9.19	77.11	7.92	1.61	1.62	1.99	1.70	1.24	1.35
US Born	76.67	8.85	76.77	8.66	75.77*	8.54	1.63	1.78	2.04	1.82	1.30	1.48

* *p* < 0.05;

** *p* < 0.01;

*** *p* < 0.001.

**Table 8:** Impact of demographic covariates on social emotional learning environment factors.

	Climate						Bullying						SNE					
	Year 1		Year 2		Year 3		Year 1		Year 2		Year 3		Year 1		Year2		Year3	
	*M*	*SD*	*M*	*SD*	*M*	*SD*	*M*	*SD*	*M*	*SD*	*M*	*SD*	*M*	*SD*	*M*	*SD*	*M*	*SD*
Grade																		
6^th^	60.48**	12.31	68.26***	12.33	70.01***	12.71	23.19	5.61	25.87	5.65	27.55	5.83	20.32***	5.27	20.49***	5.49	21.84***	5.70
7^th^	57.49	11.40	60.99	11.74	65.83	12.91	22.85*	5.58	24.70**	5.69	26.93	5.65	17.74	5.67	19.35	4.86	20.34	5.43
8^th^	59.92	11.77	62.75	10.67	64.85	11.76	24.01	5.65	25.39	5.22	26.80	5.59	18.34	5.16	18.48	5.08	20.36	5.01
Gender																		
Male	59.94	12.01	64.71	11.53	67.34	12.09	23.60	5.60	25.50	5.46	27.05	5.26	18.95	5.33	19.48	4.93	20.91	5.13
Female	58.62	11.79	63.50	12.59	67.38	13.33	22.98	5.63	25.14	5.65	27.28	6.13	18.82	5.67	19.51	5.53	21.06	5.81
Classification																		
None	58.14	11.47	63.23	11.83	66.56	12.72	23.08	5.66	25.10	5.66	27.09	5.74	18.39	5.41	18.93	5.09	20.46	5.41
Support	63.43***	12.57	67.05***	12.36	69.54**	12.53	24.09***	5.45	26.06*	5.14	27.37	5.67	20.66***	5.46	21.31***	5.22	22.41***	5.44
Country of Origin																		
Not US	60.70*	11.58	65.43*	12.22	71.07***	12.44	23.80	5.58	25.95*	5.61	28.12***	5.78	19.47*	5.22	19.83	5.16	22.64***	5.08
US Born	58.87	11.99	63.76	11.99	66.41	12.63	23.15	5.63	25.15	5.53	26.92	5.68	18.71	5.57	19.40	5.23	20.56	5.51

* *p* < 0.05;

** *p* < 0.01;

*** *p* < 0.001.

### 3.2. Mechanism for change: The social–emotional learning environment model

To test the reliability of the hypothesized conceptual model on the relationship between student perceptions of the social–emotional learning (SEL) environment, disciplinary action, and academic performance across the three samples tested, we conducted a path analysis in AMOS using each year’s sample. Following our *a priori* model, we tested the impact of student perceptions of SEL Environment on academic performance by way of a path through student disciplinary action. Our SEL Environment latent variable was comprised of the Social Normative Expectations, Perceived Bullying, and Climate Survey, and our Academic Performance latent variable was comprised of the Language Arts, Math, Science, and Social Studies grades, in accordance with the *a priori* model’s goals. As preliminary findings did not suggest a consistent pattern across measures or time, and due to the low variability of some factors (e.g., overrepresentation of 70% or more), demographic factors can be further explored in future research as part of individual level analysis rather than in the context of the hypothesized mechanism for change.

**Figure 1:**
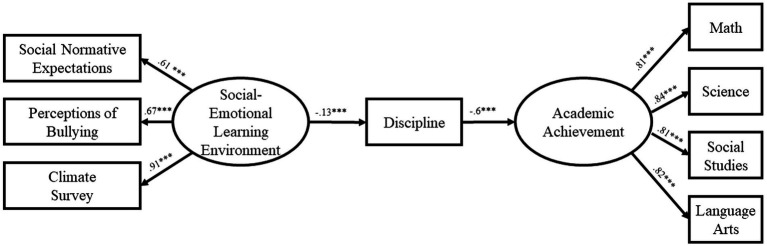
Model at year 1.

**Figure 2:**
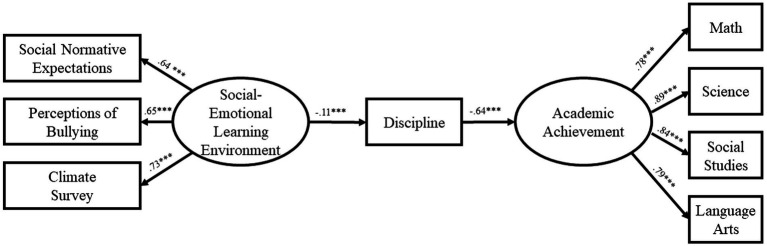
Model at year 2.

**Figure 3:**
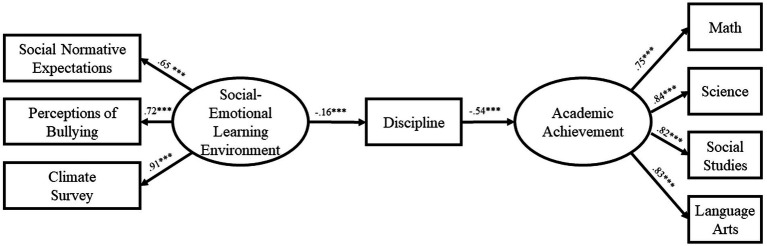
Model at year 3.

The path model for Year 1 of the study (Figure 1) demonstrated excellent fit [*χ^2^*(19) = 76.16, *CFI* = 0.99, *RMSEA* = 0.05, *TLI* = 0.98]. The effect of SEL Environment on discipline was significant (*β* = −0.13, *p* < 0.001) as was the effect of discipline on Academic Performance (*β* = −0.60, *p* < 0.001). Most importantly, the indirect effect of SEL Environment on Academic Performance was significant, if small (*β* = 0.08, *p* < 0.001). The path model for Year 2 of the study (Figure 2) likewise demonstrated excellent fit [*χ^2^*(19) = 70.68, *CFI* = 0.99, *RMSEA* = 0.048, *TLI* = 0.98]. The effect of SEL Environment on discipline was significant (*β* = −0.11, *p* < 0.001) as was the effect of discipline on Academic Performance (*β* = −0.64, *p* < 0.001). As in the first year, the indirect effect of SEL Environment on Academic Performance was significant (*β* = 0.07, *p* < 0.001). Finally, the path model for Year 3 of the study (Figure 3) also demonstrated excellent fit [*χ^2^*(19) = 66.59, *CFI* = 0.99, *RMSEA* = 0.05, *TLI* = 0.98]. The effect of SEL Environment on discipline was significant (*β* = −0.16, *p* < 0.001) as was the effect of discipline on Academic Performance (*β* = −0.54, *p* < 0.001). Once again, the indirect effect of SEL Environment on Academic Performance was significant (*β* = 0.09, *p* < 0.001). The consistency of the model across the 3 years supports the proposed relationship between SEL Environment, Discipline, and Academic Performance.

**Figure 4:**
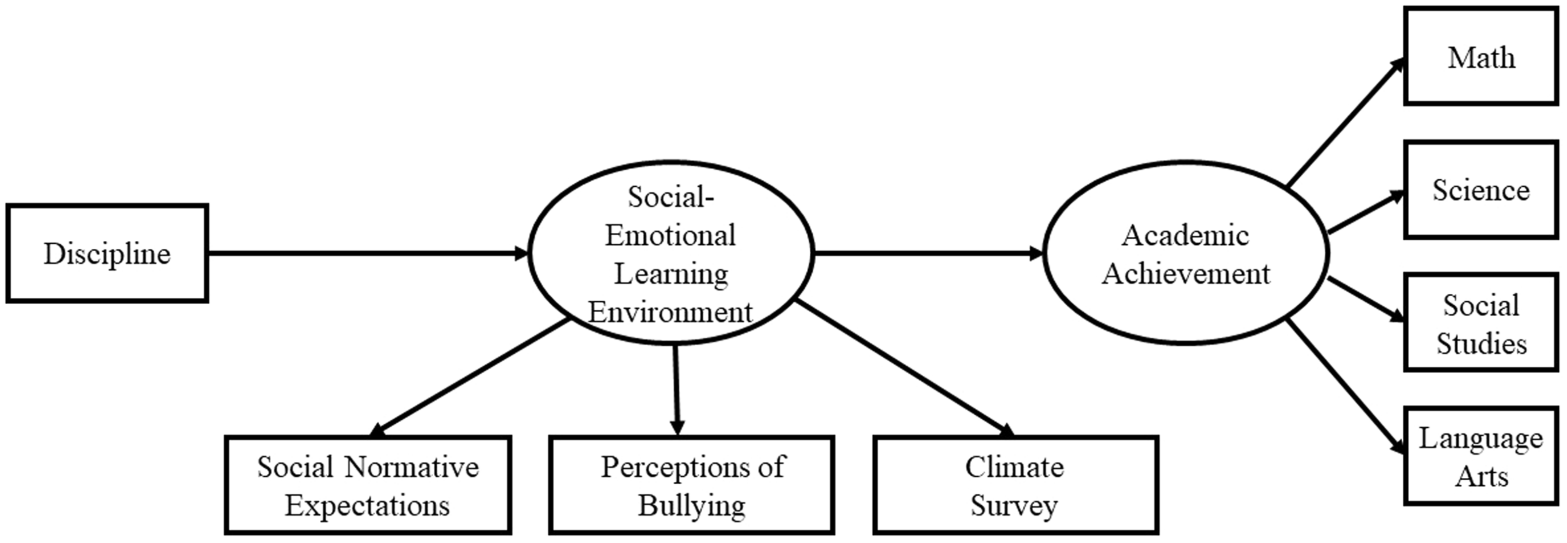
Proposed alternative model.

Best practices in SEM recommend contrasting a path analysis model with an alternative model using the same data but based on competing theories or alternative explanations. Based upon the literature reviewed here, we developed an alternative model from that of the School of Character intervention, which predicted academic performance as a result of disciplinary action mediated by student perceptions of the social–emotional learning environment (Figure 4). Fit was worse in the alternative model across all three time periods; Time 1 exhibited poor fit [*χ^2^*(57) = 1081.86, *CFI* = 0.87, *RMSEA* = 0.09, *TLI* = 0.81], Time 2 exhibited the worst fit of any model in the study [*χ^2^*(57) = 1258.2, *CFI* = 0.86, *RMSEA* = 0.09, *TLI* = 0.79], and Time 3 had the best fit of the alternative models, but still showed worse fit than our theoretical model [*χ^2^*(57) = 734.48, *CFI* = 0.9, *RMSEA* = 0.08, *TLI* = 0.86]. Given these results, we can conclude that our initial model is a better fitting model than the alternative model.

## 4. Discussion

The present study evaluated a theoretical model of change that hypothesized a relationship between student perception of social–emotional learning environment, discipline and academic achievement. Our results found that the relationships among the constructs are significant and directionally appropriate to provide support for a mechanism of change. Our data suggests that student perceptions of the social–emotional learning environment impact their disciplinary behaviors which impacts their academic achievement. Further, our results do not support an alternative theory that places disciplinary behavior as the first in the mediational cascade lending further support to the mechanism as proposed. Path analysis for each year demonstrated excellent fit ([Year 1: *χ^2^*(19) = 76.16, *CFI* = 0.99, *RMSEA* = 0.05, *TLI* = 0.98; Year 2: *χ^2^*(19) = 70.68, *CFI* = 0.99,*RMSEA* = 0.048, *TLI* = 0.98; Year 3: *χ^2^*(19) = 66.59, *CFI* = 0.99, *RMSEA* = 0.05, *TLI* = 0.98]. Further, the effect of the social–emotional learning environment construct on discipline was significant during each of the 3 years, as was the effect of discipline on Academic Performance. Finally, the indirect effect of student perceptions of the social–emotional learning environment on Academic Performance was significant across all years. The consistency of the model across the 3 years supports the proposed relationship between student perceptions of SEL Environment, Discipline, and Academic Performance.

These findings suggest that the logic model behind the School of Character Intervention, which proposed a relationship between student perceptions of social–emotional learning environment, student discipline and academic achievement, broadly held true and holds the potential to be an area to target as a mechanism for change. This model of change was implemented as an intervention in a “failing” middle school and the theory hypothesized by the intervention program was that improvement to the school as a whole begins through a positive shift in school culture and climate, and that student perceptions of the social–emotional learning environment has an impact on behavior as evidenced by disciplinary referrals. The School of Character intervention proposed that the mechanism for change proceeded along this pathway to result in student academic achievement outcomes. The current study found, in a cross-sectional analysis of each year, that the relationships between the variables proposed by the theorized logical model were related as hypothesized.

### 4.1. Limitations

This study faced several limitations that must be considered. The sample utilized may impact generalizability as it reflects a single school. The school district also had one of the lowest graduation rates in the state of New Jersey (under 60%) and reading and math testing scores ranking below the 15^th^ percentile, suggesting a particularly high needs sample. Further, the sample reflects particular demographic characteristics (e.g., majority Latinx, majority of lower SES as evidenced by over 80% of students qualifying for free lunch). As preliminary findings did not suggest a consistent pattern across measures or time, and due to the low variability of some factors (e.g., overrepresentation of 70% or more), exploration into the role of demographic factors was limited. Finally, the current study does not address the intervention itself, only the theory of the proposed change model. As a result, the current study cannot provide evidence for a causal link between the social–emotional learning environment, discipline and academic achievement, nor can we validate that changing student perceptions of the social–emotional learning environment will invariably result in a change in academic achievement. Our findings use the available data to identify the relationships between the constructs targeted in an intervention, but do not provide longitudinal or causal evidence for the efficacy of the mechanism of change itself. In light of these limitations, the results here can be considered a first step towards further research to support the intervention implications, particularly individual level analysis to test the efficacy of hypothesized mechanism for change across a range of settings.

### 4.2. Future research

The current study tested a hypothesized conceptual model that theorized a specific mechanism for change in an urban middle school: that improved academic achievement can occur as a function of perceptions of social–emotional environment and disciplinary experiences. The results are promising, as the logic model was found to be supported, with constructs relationally linked in a valid path model. The context in which this conceptual model for change was evaluated reflected a “high needs” population, thus, any factors that influence students’ achievement outcomes may present a valuable next step in resilience research. The results of this study also suggest that future research would benefit from expanded exploration of interventions targeted at these factors. If perceptions of climate, bullying and social expectations impact behavior, and which then impacts academic achievement, it may be that this relationship represents an area of resilience that can be enhanced deliberately by intervention programing that is coordinated with the elements of the model and evaluated more explicitly in sequence. To fully test the efficacy of school-level intervention programs, further research must occur in a range of schools over a number of years to see if systems level change can be executed through the path mechanism identified here. In an era when both student mental health and academic achievement are in a state of distress due to global factors beyond an individual students’ control, it is important to understand what can support our students in reaching their potential.

## Data availability statement

The original contributions presented in the study are included in the article/Supplementary material, further inquiries can be directed to the corresponding author.

## Ethics statement

The studies involving human participants were reviewed and approved by Rutgers University Institutional Review Board. Written informed consent from the participants’ legal guardian/next of kin was not required to participate in this study in accordance with the national legislation and the institutional requirements.

## Author contributions

ME contributed to conception and design of the study. GW, DH, and EV contributed to the implementational and data collection, as well as the organization of the database. GW and CS performed the statistical analyses. GW wrote the first draft of the manuscript. DH, EV, and ME contributed significantly to its revisions. MY and AW contributed to the literature review and revisions. MY and CS both wrote sections of the manuscript. All authors contributed to the article and approved the submitted version.

## Conflict of interest

The authors declare that the research was conducted in the absence of any commercial or financial relationships that could be construed as a potential conflict of interest.

## Publisher’s note

All claims expressed in this article are solely those of the authors and do not necessarily represent those of their affiliated organizations, or those of the publisher, the editors and the reviewers. Any product that may be evaluated in this article, or claim that may be made by its manufacturer, is not guaranteed or endorsed by the publisher.
