# Supplementary material for: Mechanisms for change: A theoretical pathway for a school-wide social–emotional learning initiative in an urban middle school
Source: Front Psychol. 2023 Feb 15;14:977680. doi: 10.3389/fpsyg.2023.977680 (PMC9975162; doi:10.3389/fpsyg.2023.977680)
Supplement: Supplementary file 1 [file Data_Sheet_1.docx]

**Supplementary Online Materials**

**Study Measures**

**Social Normative Expectations:**

Please rate how much you agree with the following statements:

1= Disagree A LOT! 2=Disagree 3=Neither Agree nor Disagree 4=Agree 5= Agree A LOT!

1: In the future, most students from this school will graduate from high school

2: In the future, most students in this school will go to college

3: In the future, most students in this school will have a job that pays well

4: In the future, most students in this school will contribute meaningfully to our communities

5: In the future, most students in this school will have a happy family life

6: In the future, most students in this school will stay in good health most of the time

**Additional Study Results (detailed)**

During year 1, 2 and 3, Academic Achievement and discipline were consistently, significantly negatively related (year 1: *r*(1140)=-.57; year 2: *r*(1215)=-.61; *r*(1012)=-.51, p<.001 for all). The relationship between Academic Achievement and school climate was only significant during year 2 (*r*(1215)=.08, *p*=.004) and year 3 (*r*(1012)=.08, *p*=.017). The relationship between Academic Achievement and bullying was only significantly during year 3 (*r*(1012)=.08, *p*=.016) while the relationship between Academic Achievement and social normative expectations was only significant during the first year (r(1140)=-.07, *p*=.019). Across year 1, 2 and 3, the number of discipline referrals and student perceptions of school climate were consistently, significantly negatively related (year 1: *r*(1140)=-.12; year 2: *r*(1215)=-.13; *r*(1012)=-.16, *p*<.001 for all). However, discipline was only related to bullying during year 1 (*r*(1140)=-.10, *p*=.001) and year 3 (*r*(1012)=-.12, *p*<.001), and social normative expectations during year 1 (*r*(1140)=-.08, *p*=.005). Student perceptions of their school’s climate and their perceived lack of bullying were significantly positively related (year 1: *r*(1140)=.61; year 2: *r*(1215)=.47; *r*(1012)=.66, *p*<.001 for all) as were climate and social normative expectations (year 1: *r*(1140)=.56; year 2: *r*(1215)=.47; *r*(1012)=.60, *p*<.001 for all). Student perceptions of safety (absence of bullying behaviors) was significantly positively related to social normative expectations across all three years (year 1: *r*(1140)=.41; year 2: *r*(1215)=.52; *r*(1012)=.58, *p*<.001 for all).

During year 1 and year 2, grade level had a significant impact on overall achievement (*F*(2,1137)=4.98, *p*=.008; *F*(2,1212)=11.33, *p*<.001 respectively) but by year 3, no significance was found. During all years, grade level was related to number of disciplinary referrals, (*F*(2,1137)=21.28, *p*=.001; *F*(2,1212)=3.79, *p*=.023; *F*(2,1009)=3.29, *p*=.038 respectively) with 7^th^ graders having the greatest number of referrals in years 1 and 2, and 6^th^ graders in year 3 (see Table 7). Independent samples *t-*tests resulted in a consistently significant impact of gender across all three years on Academic Achievement (*t*(1138)=-8.09, *p*<.001; *t*(1213)=-7.60, *p*<.001; *t*(1010)=-9.58, *p*<.001 respectively) and discipline (*t*(1138)=3.50, *p*<.001; *t*(1213)=4.22, *p*<.001; *t*(1010)=5.88, *p*<.001 respectively) with girls evidencing better grades and fewer disciplinary referrals. Whether a student was provided educational support (IEP or LEP) did not have a consistent impact on grades and discipline, with an impact on grades only during year 2 (*t*(1213)=3.12, *p*=.002) and disciplinary referrals in year 2 and 3 (*t*(1213)=-3.82, *p*<.001; *t*(1010)=-3.96, *p*<.001 respectively). Country of birth only had an impact on grade during year 3 (*t*(1010)=2.05, *p*=.041) with students born in the US reporting a lower Academic Achievement (see Table 7).

The receipt of support via a classification (IEP or LEP) had a consistent impact on perceptions of school climate during year 1, year 2 and year 3 (*t*(1138)=-6.30, *p*<.001; *t*(1213)=-4.73, *p*<.001; *t*(1010)=-3.31, *p*=.001 respectively) and social normative expectations (*t*(1138)=-5.87, *p*<.001; *t*(1213)=-6.87, *p*<.001; *t*(1010)=-5.07, *p*=.001 respectively) with students receiving support having more positive perceptions of climate and social normative expectations. The impact of support on bullying was only significant during the first two years (*t*(1213)=-2.50, *p*=.013; *t*(1010)=-2.55, *p*=.011 respectively), again with students with a classification reporting less bullying behaviors. Being born in the United States appeared to have an impact on all SEL measures, with US born students consistently reporting a more positive perception of school climate (*t*(1138)=2.20, *p*=.028; *t*(1213)=2.00, *p*=.046; *t*(1010)=4.76, *p*<.001 respectively). The impact was less consistent for perceptions of bullying which was only impacted during year 2 and year 3 (*t*(1213)=2.10, *p*=.036; *t*(1010)=2.69, *p*=.007 respectively) and social normative expectations which only evidenced significance during year 1 and 3 (*t*(1138)=1.99, *p*=.047; *t*(1010)=4.94, *p*<.001 respectively; see **Table 8**).
